# The bZIP transcription factor ATF1 regulates blue light and oxidative stress responses in *Trichoderma guizhouense*

**DOI:** 10.1002/mlf2.12089

**Published:** 2023-12-04

**Authors:** Yifan Li, Yanshen Li, Huanhong Lu, Tingting Sun, Jia Gao, Jian Zhang, Qirong Shen, Zhenzhong Yu

**Affiliations:** ^1^ Nanjing Agricultural University, Jiangsu Provincial Key Lab for Organic Solid Waste Utilization, National Engineering Research Center for Organic‐based Fertilizers, Jiangsu Collaborative Innovation Center for Solid Organic Waste Resource Utilization Agricultural Microbial Resources Protection and Germplasm Innovation and Utilization Center of Jiangsu Province Nanjing China; ^2^ Department of Microbiology Karlsruhe Institute of Technology (KIT)—South Campus, Institute for Applied Biosciences Karlsruhe Germany

**Keywords:** bZIP transcription factor ATF1, light responses, MAPK HOG1, oxidative stress, *Trichoderma guizhouense*

## Abstract

In several filamentous fungi, incident light and environmental stress signaling share the mitogen‐activated protein kinase (MAPK) HOG (SAK) pathway. It has been revealed that short‐term illumination with blue light triggers the activation of the HOG pathway in *Trichoderma* spp. In this study, we demonstrate the crucial role of the basic leucine zipper transcription factor ATF1 in blue light responses and signaling downstream of the MAPK HOG1 in *Trichoderma guizhouense*. The lack of ATF1 severely impaired photoconidiation and delayed vegetative growth and conidial germination. Upon blue light or H_2_O_2_ stimuli, HOG1 interacted with ATF1 in the nucleus. Genome‐wide transcriptome analyses revealed that 61.8% (509 out of 824) and 85.2% (702 out of 824) of blue light‐regulated genes depended on ATF1 and HOG1, respectively, of which 58.4% (481 out of 824) were regulated by both of them. Our results also show that blue light promoted conidial germination and HOG1 and ATF1 played opposite roles in controlling conidial germination in the dark. Additionally, the lack of ATF1 led to reduced oxidative stress resistance, probably because of the downregulation of catalase‐encoding genes. Overall, our results demonstrate that ATF1 is the downstream component of HOG1 and is responsible for blue light responses, conidial germination, vegetative growth, and oxidative stress resistance in *T. guizhouense*.

## Impact statement

In fungi, the MAPK HOG pathway responsible for environmental stress responses plays a pivotal role in light signaling. Here, we demonstrate that the bZIP transcription factor ATF1 functions the downstream of the HOG pathway in response to blue light in *Trichoderma guizhouense*. ATF1 interacts with HOG1 in the nucleus upon blue light stimulus to control a large proportion of the light‐regulated genes. In addition, ATF1 controls photoconidiation, conidial germination, vegetative growth, and stress resistance. Therefore, ATF1 is crucial for light responses and signaling in *T. guizhouense*.

## INTRODUCTION

In multicellular eukaryotes, basic leucine zipper (bZIP) transcription factors (TFs) participate in signal transduction pathways to regulate cellular homeostasis, organism development, and responses to environmental stresses^1^, ^2^. The fungal bZIP TF Atf1 was first identified in *Schizosaccharomyces pombe* in the 1990s. Since then, various functions of its orthologs in other fungi have been characterized. These TFs are typically involved in the regulation of vegetative growth, development, stress resistance, secondary metabolism, and virulence^2^, ^3^. For instance, in the filamentous fungus *Aspergillus nidulans*, the absence of the Atf1 ortholog AtfA promotes sexual development^4^, ^5^, ^6^, and conidia of the *ΔatfA*‐mutant strain show increased sensitivity to osmotic, oxidative, and heat stress and to fungicide^5^, ^7^, ^8^. In the human pathogen *Aspergillus fumigatus*, the deletion of AtfA results in decreased virulence, accompanied by reduced vegetative growth and conidiospore viability^9^, ^10^, ^11^.

In fungi, the mitogen‐activated protein kinase (MAPK) signaling pathway is involved in responses to various environmental signals, including light and a series of abiotic stresses^5^, ^12^, ^13^, ^14^, ^15^. In *S. pombe*, the expression level of *atf1* is regulated by the MAPK Sty1 in response to osmotic, oxidative, heat shock, and starvation stresses^16^, ^17^. The phosphorylated MAPK Sty1 associates with Atf1 in the nucleus under various stress conditions^18^, ^19^. In *A. nidulans* and *A. fumigatus*, the MAPK SakA is phosphorylated in response to oxidative and osmotic stress stimuli and then interacts with AtfA in the nucleus to modulate gene expression^5^, ^20^, ^21^. In *A. nidulans*, the MAPK SakA signaling pathway is also activated in light, and ~98% of the light‐regulated genes are controlled by SakA^22^. However, the regulatory role of AtfA in response to light has not been studied at the genome‐wide level. Nonetheless, AtfA in *A. nidulans* is known to regulate the expression of the light‐inducible genes *ccgA* and *conJ* ^13^. The regulatory roles of AtfA orthologs in light responses in *A. nidulans* and other fungi are yet to be elucidated.

*Trichoderma* species exhibit remarkable abilities to promote plant growth and strengthen plant defenses against various biotic and abiotic stresses. For these reasons, *Trichoderma*‐based biocontrol agents are widely used in agriculture^23^, ^24^, ^25^. Photobiology studies in *Trichoderma* have shown that blue light can efficiently promote conidia production and increase the resistance of conidia to several environmental stresses. The blue light receptor BLR1, the ortholog of *Neurospora crassa* white collar‐1 protein, tightly controls the photoconidiation process^26^, ^27^, ^28^, ^29^. Recent genome‐wide transcriptome analyses have revealed that BLR1 is the main component of the blue light signaling network in *Trichoderma*.^28^, ^29^. The ENVOY (ENV1) protein, an ortholog of the blue light receptor VIVID of *N. crassa*, is critical for repressing BLR1 activity to achieve photoadaptation^28^, ^29^, ^30^, ^31^. In *Trichoderma*, blue light can induce the gene expression and the phosphorylation of the MAPK Tmk3/HOG1 through BLR1^28^, ^32^. Our previous study in *Trichoderma guizhouense* also demonstrated that the MAPK HOG1 translocates from the cytoplasm into the nucleus upon blue light stimulus, consistent with the finding in *A. nidulans* that SakA (HogA) accumulates in the nucleus after red light exposure^13^, ^28^. Additionally, the MAPK HOG1 controls a large proportion of blue light‐regulated genes, including stress‐related genes encoding catalases, photolyases, heat shock proteins, and hydrophobins^28^, ^33^.

In *Trichoderma* species, the orthologs of Atf1/AtfA have not been identified so far, and their roles in the light signaling network are still unknown. In this study, we demonstrate the involvement of ATF1 in conidial germination, vegetative growth, photoconidiation, and stress resistance in *T. guizhouense*. Our results show that ATF1 physically interacts with HOG1 in the nucleus under blue light or oxidative stress conditions. The results of genome‐wide transcriptome analyses further reveal that ATF1 regulates more than 60% of the differentially expressed genes (DEGs) identified in the wild type upon short‐term blue light exposure and prove that ATF1 in association with HOG1 plays crucial roles in blue light responses.

## RESULTS

### Blue light regulates *atf1* expression through the blue light receptor BLR1 and the MAPK HOG1 in *T. guizhouense*

To identify the ortholog of Atf1/AtfA in *T. guizhouense*, we performed a BLAST search against the genome with the protein sequence of AtfA from *A. nidulans* as the query. This search identified a putative bZIP TF (OPB40254) with high similarity to *A. nidulans* AtfA (sequence identity: 52%; *e*‐value: 5e−114). Analyses using tools at the Conserved Domain Database (CDD) revealed that OPB40254 contained four conserved structural domains: OSA, HRA, HRR, and ATF‐2 (Figure S1A,B). Therefore, the gene OPB40254 was named *atf1*.

**Figure 1:**
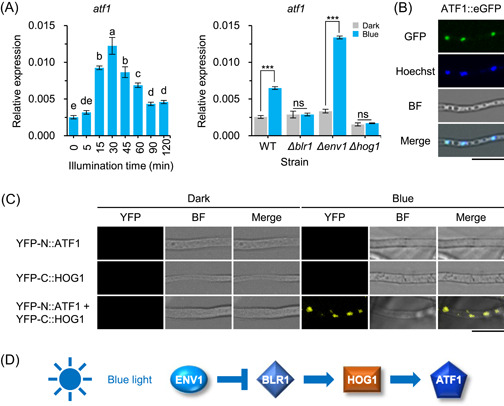
Transcript profile of *atf1* under blue light. (A) Changes in transcript levels of *atf1* in response to different blue light exposures (left) and different strains (right). Left: the wild type was grown on potato dextrose agar (PDA) medium at 28°C in the dark for 24 h before exposure to blue light for 5, 15, 30, 45, 60, 90, and 120 min. Data were analyzed by one‐way analysis of variance with Bonferroni correction. Different lowercase letters represent significant differences (*p* < 0.05). Right: Transcript levels of *atf1* in different strains. Wild type and *Δblr1*‐, *Δenv1*‐, and *Δhog1*‐mutant strains were cultured for 24 h and then exposed to blue light for 30 min. Transcript level of each gene was normalized to that of *tef1*. Error bars indicate standard deviation (SD) of three biological replicates. Data were analyzed by *t*‐test (***p < 0.001, ns, not significant). (B) Subcellular localization of ATF1. Fresh conidia of ATF1::eGFP strain were incubated on microscope coverslips with 400 µl PDB in the dark for 14 h. Samples were then stained with Hoechst. Scale bar, 10 μm. (C) Visualization of interaction between ATF1 and HOG1 upon blue light exposure using BiFC. Strains were grown on PDA medium in the dark for 24 h and then exposed to blue light for 3 min. Scale bar, 10 μm. (D) Schematic diagram of activation of ATF1 by blue light in *Trichoderma guizhouense*. WT, wild type; YFP, yellow fluorescent protein.

To analyze the impact of blue light on the transcription profile of *atf1*, the transcript levels of *atf1* in response to light for different exposure periods were measured by quantitative real‐time PCR (RT‐qPCR) in the wild type. The transcript level of *atf1* increased significantly after 15 min of illumination and peaked at 30 min, and then decreased and leveled off after 90 min of illumination (Figure 1A). To explore whether this light‐regulated expression of *atf1* depends on the photoreceptors BLR1 and ENV1 and the MAPK HOG1, the transcript levels of *atf1* were measured by RT‐qPCR in the wild type and the *Δblr1*‐, *Δenv1*‐, and *Δhog1*‐mutant strains after 30 min of illumination. The transcript level of *atf1* increased by 2.5‐fold in the wild type upon blue light exposure, but was not upregulated by blue light in the *Δblr1*‐ and *Δhog1*‐mutant strains (Figure 1A). However, compared with darkness, under blue light, the *atf1* transcript level in the *Δenv1*‐mutant strain was increased by 4.0‐fold. In addition, after blue light exposure, the *atf1* transcript level in the *Δenv1*‐mutant strain was 2.1‐fold that in the wild type. This result suggests an essential role of BLR1 and HOG1 in blue light regulation of *atf1* expression and that ENV1 negatively regulates *atf1* expression.

### Interaction between ATF1 and HOG1 is activated by blue light

To show whether ATF1 is a nuclear protein in *T. guizhouense*, we constructed a strain expressing an eGFP‐tagged ATF1 fusion protein. In this strain, eGFP signals were observed exclusively in nuclei (Figure 1B). We further investigated the physical interaction between HOG1 and ATF1 in response to blue light with the bimolecular fluorescence complementation (BiFC) assays. Three strains individually expressing ATF1 fused with the N‐terminal half of yellow fluorescent protein (YFP‐N‐ATF1), HOG1 fused with the C‐terminal half of YFP (YFP‐C‐HOG1), and both fusion proteins under the control of the *cdna1*‐gene promoter were constructed. All strains were cultured on potato dextrose agar (PDA) plates in the dark for 24 h and then exposed to blue light for 3 min. Two control strains harboring only half of the YFP sequence did not produce fluorescence signals either in the dark or under blue light. In the strain expressing both fusion proteins, YFP signals appeared after the blue light stimulus, whereas no fluorescence was detected in the dark (Figure 1C). Therefore, HOG1 translocates into the nucleus to activate ATF1 upon blue light illumination in *T. guizhouense* (Figure 1D).

### ATF1 regulates photoconidiation and mycelial growth in *T. guizhouense*

**Figure 2:**
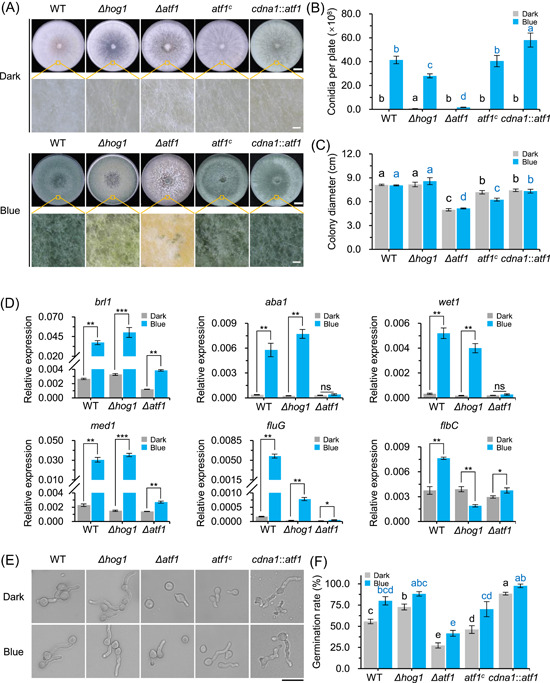
Impact of HOG1 and ATF1 on the photoconidiation process in *Trichoderma guizhouense*. (A) Phenotype analysis of wild type and mutant strains under light and dark conditions. Mycelial mats (4 mm) of wild type and *Δhog1*‐, *Δatf1*‐, *atf1* ^*c*^‐, and *cdna1*::*atf1*‐mutant strains were inoculated onto potato dextrose agar (PDA) plates and grown for 3 days. Colony surface was observed and photographed under a stereoscopic microscope. Scale bars, 1 cm (upper panels) and 300 μm (lower panels). (B) Amount of conidia produced by each strain under dark and light conditions after 3 days of culture. (C) Growth rates of each strain under blue light and dark conditions. Mycelial mat (4 mm) of each strain was inoculated onto a PDA plate and grown in the dark or under blue light, and colony diameter was measured after 48 h of culture. (D) Transcript levels of genes essential for conidiation in wild type and *Δhog1*‐ and *Δatf1*‐mutant strains. All strains were cultured in the dark or under blue light for 48 h. Transcript level of each gene was normalized to that of *tef1*. Data were analyzed by *t*‐test (*p < 0.05, **p < 0.01, ***p < 0.001, ns, not significant). (E) Micrographs showing germination status of wild‐type and mutant strains under light and dark conditions. Wild type and *Δhog1*‐, *Δatf1*‐, *atf1* ^*c*^‐, and *cdna1*::*atf1*‐mutant strains were grown in PDB medium for 12 h. Scale bar, 20 μm. (F) Germination rate of conidia after 12 h of culture. Error bars indicate SD of three biological replicates. Data were analyzed by one‐way analysis of variance with Bonferroni correction. Lowercase letters of different colors (black, dark condition; blue, blue light condition) represent significant difference (B, C, F; *p* < 0.05).

To further investigate the biological function of ATF1 in the light responses of *T. guizhouense*, *atf1* was replaced by homologous recombination with the hygromycin‐resistance gene *hph* as the selective marker. Three positive transformants with identical phenotypes were obtained and verified by diagnostic PCR and Southern blot analysis (Figure S2A–D). A re‐complemented strain *atf1* ^*c*^ was constructed by introducing the full‐length *atf1* gene back into the mutant. The mutant strain *cdna1*::*atf1* with an overexpression allele of ATF1 was also constructed, and the high transcript level of *atf1* was verified by RT‐qPCR (Figure S2E). Indeed, blue light induced *hog1* expression in the absence of ATF1 but did not induce *atf1* expression in the absence of HOG1, providing evidence that ATF1 functions downstream of HOG1 (Figure S2F). We then analyzed conidia production in the different strains in the dark and under blue light. In blue light, the wild type produced large quantities of green conidia, while the deletion of *atf1* led to an almost complete loss of conidia formation (Figure 2A,B). In the re‐complemented strain *atf1* ^*c*^, photoconidiation was recovered. In the *cdna1*::*atf1*‐mutant strain, the conidia yield was a bit more higher than that of the wild type under blue light. In agreement with our previous study^28^, the *Δhog1*‐mutant strain also formed green conidia under blue light, but the conidia yield was lower than that of the wild type. The mycelial growth rate of the strains on PDA plates was compared (Figure 2C). The *Δatf1*‐mutant strain grew slower than the wild‐type strain, the *Δhog1*‐mutant strain, the re‐complemented strain *atf1* ^*c*^, and the *cdna1*::*atf1*‐mutant strain under both dark and light conditions. Notably, despite numerous attempts, the double deletion mutant (*Δhog1Δatf1*) could not be generated. This suggests that there may be a synthetic lethal genetic interaction between the two genes and supports the nonoverlapping cellular functions of HOG1 and ATF1.

Next, we measured the transcript levels of conidiation‐related genes (*brl1*, *aba1*, *wet1*, *med1*, *fluG*, and *flbC*) by RT‐qPCR in the wild type and the *Δhog1*‐ and *Δatf1*‐mutant strains after 48 h of culture in constant blue light. In the wild type, the transcript levels of these genes were markedly higher under blue light than in darkness (Figure 2D). The transcript levels of *brl1*, *aba1*, *wet1*, and *med1* genes in the *Δhog1*‐mutant strain under blue light were similar to those in the wild type, consistent with our previous finding that the deletion of *hog1* did not block the induction of these genes by blue light^28^. However, in the *Δatf1*‐mutant strain, the transcript levels of *aba1* and *wet1* did not increase upon blue light illumination. While the transcript levels of *brl1*, *med1*, *flbC*, and *fluG* in the *Δatf1*‐mutant strain increased under blue light, they were still significantly lower than those in the wild type. These results suggest that ATF1 plays a crucial regulatory role in light‐regulated conidiogenesis.

### ATF1 and HOG1 contribute to conidial germination independent of blue light exposure

In the plant pathogenic fungus *Alternaria alternata*, light inhibits conidial germination, and the MAPK HogA plays a repressive role in this process^34^. To assess the effect of blue light on conidial germination in *T. guizhouense*, 1 × 10^5^ conidia of the wild type and the *Δhog1*‐, *Δatf1*‐ *atf1* ^*c*^‐, and *cdna1*::*atf1*‐mutant strains produced under blue light were cultured on coverslips with 400 µl PDB medium in the dark or under blue light, and the germination rate was determined after 12 h of culture. In all tested strains, blue light significantly accelerated conidial germination (Figure 2E,F). In the wild type, 80.0% of conidia germinated under blue light after 12 h, whereas the germination rate in the dark was only 55.6%. Interestingly, in the *Δhog1*‐mutant strain, conidia germinated earlier than in the wild type in both dark and light conditions. The germination rate in the *Δhog1*‐mutant strain after 12 h reached 88.2% under blue light and 72.7% in the dark. Inversely, conidial germination was delayed in the *Δatf1*‐mutant strain—the conidial germination rate was 41.6% under blue light and only 27.3% in the dark after 12 h of culture. In the *atf1* ^*c*^‐mutant strain, the germination rate was similar to that of the wild type under both dark and light conditions. By contrast, conidia of the *cdna1*::*atf1*‐mutant strain germinated earlier than those of the wild type. Compared with the wild type, the *cdna1*::*atf1*‐mutant strain showed 59.0% and 22.0% higher conidial germination in the dark and under blue light, respectively, after 12 h of culture.

### ATF1 controls the expression of a large proportion of blue light‐regulated genes in *T. guizhouense*

**Figure 3:**
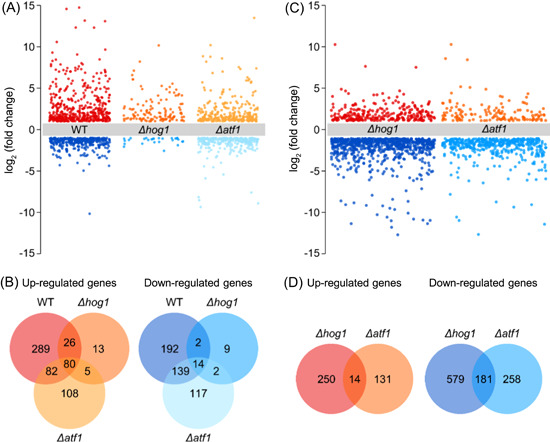
Transcriptome analysis of wild type and *Δhog1*‐ and *Δatf1*‐mutant strains upon blue light exposure. (A) Group volcano diagrams of differentially expressed genes (DEGs) in wild type (WT) and *Δhog1*‐ and *Δatf1*‐mutant strains upon blue light exposure. DEGs were screened by comparison of gene expression between dark and blue light conditions. (B) Venn diagrams of DEGs in wild type and *Δhog1*‐ and *Δatf1*‐mutant strains after blue light exposure. (C) Group volcano diagrams of DEGs in *Δhog1*‐ and *Δatf1*‐mutant strains in the dark. DEGs were screened by comparison of gene expression between mutant strain and wild type in the dark. (D) Venn diagrams of DEGs in *Δhog1*‐ and *Δatf1*‐mutant strains in the dark. DEGs were screened with the thresholds of |log_2_(fold change)|≥1.0 and false discovery rate (FDR) < 0.05.

Given the importance of ATF1 in the light responses of *T. guizhouense*, we performed transcriptome sequencing of the wild type and the *Δhog1*‐ and *Δatf1*‐mutant strains to further investigate its regulatory role in the control of light‐regulated genes. After exposure to blue light for 30 min, 824, 151, and 547 DEGs (|log_2_(fold change)|≥1, FDR < 0.05) were identified in the wild type and *Δhog1*‐ and *Δatf1*‐mutant strains, respectively (Figure 3A,B). In the wild type, 477 genes were upregulated and 347 genes were downregulated after blue light exposure: 61.8% (509 out of 824) of these DEGs were regulated by ATF1, and 94.5% (481 out of 509) of the ATF1‐dependent DEGs were also regulated by HOG1. Besides, 315 DEGs (162 upregulated and 153 downregulated) identified in the wild type were also differentially expressed in the *Δatf1*‐mutant strain, and 70.2% (221 out of 315) of these DEGs were regulated by HOG1. In the *Δatf1*‐mutant strain, 275 upregulated and 272 downregulated genes were identified after blue light exposure. Among them, 232 genes (113 upregulated and 119 downregulated) were not differentially expressed in the wild type, and only seven of them (five upregulated and two downregulated) were also differentially expressed in the *Δhog1*‐mutant strain.

Next, we compared gene expression between the mutant strains and the wild type in the dark. In the *Δatf1*‐mutant strain, 145 genes were upregulated compared with the wild type, and only 14 of them (9.7%) were also upregulated in the *Δhog1*‐mutant strain (264 upregulated) (Figure 3C,D). However, more genes were repressed in the *Δatf1*‐mutant strain. 439 downregulated genes were identified in the *Δatf1*‐mutant strain, and 181 of them (41.2%) overlapped with the downregulated genes in the *Δhog1*‐mutant strain (760 downregulated). Gene Ontology (GO) enrichment analyses were performed for the DEGs in the dark (Figure S3A). In both mutants, the top three GO terms enriched with genes upregulated in the dark were “catalytic activity,” “metabolic process,” and “single‐organism process.” The GO terms enriched most significantly with downregulated genes were the same as those enriched with upregulated genes. Kyoto Encyclopedia of Genes and Genomes (KEGG) pathway enrichment analyses of DEGs in each mutant were performed, and the top 10 enriched pathways (FDR < 0.05) are listed in Figure S3B. The absence of HOG1 and ATF1 affected many aspects of fungal metabolism, such as amino acid metabolism, fatty acid metabolism, lipid metabolism, carbon metabolism, and nitrogen metabolism.

### Transcription factor genes are regulated by ATF1 in *T. guizhouense*

**Figure 4:**
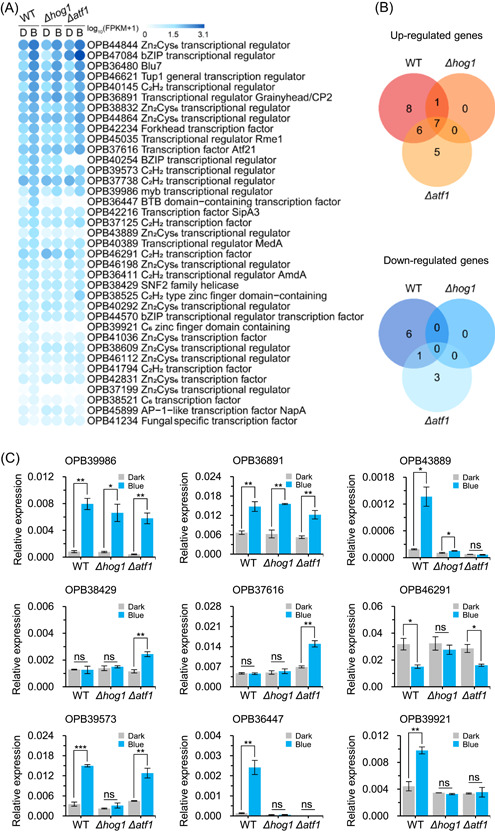
Transcript profiles of genes encoding transcription factors (TFs) under blue light. (A) Transcript profiles of blue light‐regulated TF‐encoding genes screened from the differentially expressed genes. D and B represent dark and blue light conditions, respectively. (B) Venn diagrams of blue light‐regulated TF‐encoding genes in wild type and *Δhog1*‐ and *Δatf1*‐mutant strains. (C) Transcript levels of selected TF‐encoding genes in wild type and *Δhog1*‐ and *Δatf1*‐mutant strains. All strains were grown on potato dextrose agar medium at 28°C in the dark for 24 h before exposure to blue light for 30 min. Transcript level of each gene was normalized to that of *tef1*. Error bars indicate SD of three biological replicates. Data were analyzed by *t*‐test (*p < 0.05, **p < 0.01, ***p < 0.001, ns, not significant).

In the transcriptome analysis, 37 TF‐encoding genes were identified among the total 1078 DEGs from all strains under blue light, including 27 upregulated and 10 downregulated ones (Figure 4A,B). In the wild type, 22 and seven TF‐encoding genes were upregulated and downregulated, respectively. Among them, 15 genes were controlled by ATF1, and 14 of the ATF1‐regulated genes were also controlled by HOG1. Additionally, eight TF‐encoding genes were only differentially expressed in the *Δatf1*‐mutant strain, and seven TF‐encoding genes that were differentially expressed in the wild type depended on neither HOG1 nor ATF1. The transcript profiles of these genes were confirmed by RT‐qPCR (Figure 4C).

Notably, in the dark, the two mutant strains had 50 TF‐encoding genes among their DEGs compared with WT (Figure S4A,B). Among them, 13 genes were upregulated in the *Δhog1*‐mutant strain and two were upregulated in the *Δatf1*‐mutant strain. Fourteen TF‐encoding genes were downregulated in the *Δatf1*‐mutant strain, and five of them overlapped with the downregulated genes in the *Δhog1*‐mutant strain (27 downregulated). The transcript profiles of these genes were confirmed by RT‐qPCR (Figure S4C).

### ATF1 is required for oxidative stress resistance in *T. guizhouense*

**Figure 5:**
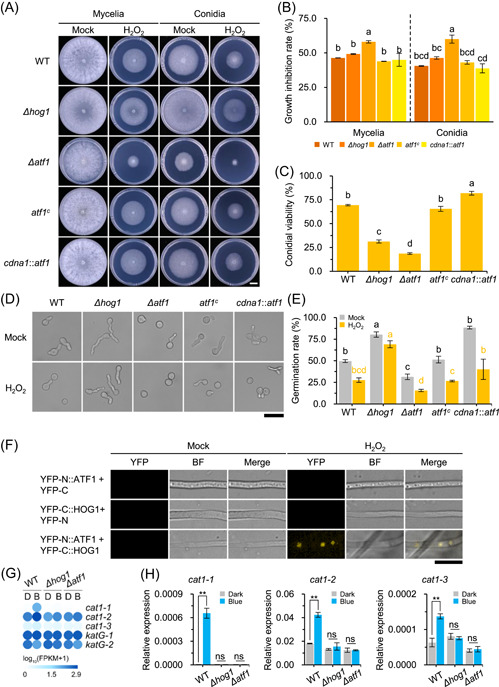
Effect of HOG1 and ATF1 on oxidative stress resistance in *Trichoderma guizhouense*. (A) Oxidative stress resistance of mycelia and conidia of each strain. All strains were cultured on PDA medium containing 30 mM (mycelia) or 3 mM H_2_O_2_ (conidia) or not at 28°C in the dark. Phenotype of each strain was photographed. Scale bar, 1 cm. (B) Growth inhibition rate of each strain was calculated after 2 days of culture. (C) Conidia viability of each strain under oxidative stress conditions. Conidia were spread on PDA medium with or without 3 mM H_2_O_2_, and their viability was calculated after 30 h incubation in the dark. (D) Phenotype of conidia of each strain under oxidative stress conditions. Conidia of each strain were inoculated on PDB medium with or without 1.5 mM H_2_O_2_ and cultured at 28°C in the dark for 12 h. Scale bar, 20 μm. (E) Conidial germination rate of each strain was measured after 12 h culture. One‐way analysis of variance (ANOVA) with the Bonferroni correction was used for statistical analysis, and lowercase letters of different colors (black, dark condition; orange, H_2_O_2_ stress condition) represent significant difference (B, C, E; *p* < 0.05). (F) BiFC analysis of interaction between ATF1 and HOG1 after H_2_O_2_ stimulus. Strains were grown on PDA medium in the dark for 24 h and then treated with 10 mM H_2_O_2_ for 3 min. Scale bar, 10 μm. (G) Transcriptional abundance of catalase (CAT)‐encoding genes *cat1‐1* (OPB39159), *cat1‐2* (OPB42210), *cat1‐3* (OPB40299), *katG‐1* (OPB43868), and *katG‐2* (OPB40552) in each strain under dark and blue light conditions derived from transcriptome data. For each gene, a FPKM (fragments per kilobase of transcript per million mapped reads) value was calculated to quantify its transcript abundance and variation. D and B represent dark and blue light conditions, respectively. (H) Transcript levels of *cat1‐1*, *cat1‐2*, and *cat1‐3* in wild type and *Δhog1*‐ and *Δatf1*‐mutant strains. All strains were grown on PDA medium at 28°C in the dark for 24 h before exposure to blue light for 30 min. Transcript level of each gene was normalized to that of *tef1*. Data represent mean ± SD (*n* = 3). Data were analyzed by *t*‐test (**p < 0.01, ns, not significant).

In *A. nidulans*, SakA (HogA) and AtfA are required for the oxidative stress resistance of conidia^5^, ^35^. To evaluate the effects of HOG1 and ATF1 on oxidative stress resistance in *T. guizhouense*, fresh mycelia or conidia of the wild type and the *Δhog1*‐, *Δatf1*‐, *atf1* ^*c*^‐, and *cdna1*::*atf1*‐mutant strains were inoculated onto PDA plates in the absence or presence of H_2_O_2_, and the growth inhibition rate of each strain was calculated after 2 days of culture in the dark. The conidia and mycelia of all strains were sensitive to H_2_O_2_, and conidia were more sensitive than mycelia (Figure 5A–C). Compared with the wild type and the *Δhog1*‐mutant strain, the *Δatf1*‐mutant strain was more sensitive to H_2_O_2_. When *atf1* was re‐complemented or overexpressed, the growth inhibition rates of the mutant strains were similar to that of the wild type. The impact of oxidative stress on conidial germination was also analyzed. In the presence of oxidative stress, conidial germination of the wild type and the *Δatf1*‐, *atf1* ^*c*^‐, and *cdna1*::*atf1*‐mutant strains was significantly delayed (Figure 5D,E). However, conidial germination of the *Δhog1*‐mutant strain was only slightly inhibited by oxidative stress. Therefore, the suppression of conidial germination by H_2_O_2_ is likely regulated by HOG1 but not by ATF1.

In *A. nidulans*, SakA physically interacts with AtfA to regulate the oxidative stress response^5^. Therefore, the interaction between HOG1 and ATF1 upon oxidative stress was evaluated in *T. guizhouense*. Indeed, the YFP signal was detected in nuclei after adding hydrogen peroxide, and no fluorescence appeared in the control strains (Figure 5F). By analyzing the transcriptome data, five catalase‐encoding genes were identified, three of which (*cat1‐1*, *cat1‐2*, and *cat1‐3*) were upregulated in the wild type upon blue light exposure (Figure 5G). This upregulation of *cat* genes was verified by RT‐qPCR analysis (Figure 5H). In the *Δhog1*‐ and *Δatf1*‐mutant strains, blue light did not affect the transcript levels of *cat1‐1*, *cat1‐2*, and *cat1‐3*.

## DISCUSSION

Light regulates important physiological and morphological processes of fungi^36^, ^37^, ^38^, ^39^, ^40^. Blue light can trigger photoconidiation of *T. guizhouense*, and this process relies on the blue light receptor BLR1^28^, ^33^. The MAPK HOG signaling pathway is also involved in photoconidiation. In the *Δblr1*‐mutant strain, photoconidiation was abolished, and in the *Δhog1*‐mutant strain, it was also impaired to some extent^28^. The orthologs of the bZIP TF ATF1 in several fungi, such as *A. nidulans* and *Botrytis cinerea*, maintain normal conidiation under moderate conditions without any stimuli^4^, ^5^, ^6^, ^41^. In this study, we proved the crucial role of ATF1 in blue light‐activated conidiation in *T. guizhouense*. The lack of ATF1 severely retarded conidiation under blue light and severely impaired the light‐induced activation of a series of conidiation‐related genes. However, the *Δhog1*‐mutant strain still produced a considerable amount of conidia under blue light, but the expression of *fluG* and *flbC* was disrupted. In this respect, ATF1 appears to be more important than HOG1. It has been reported that AtfA orthologs interact with other TFs to form heterodimers in other fungi^5^, ^21^, ^42^, ^43^. We speculate that ATF1 serves not only as a downstream component of HOG1 but also as the recipient of signals from other pathways.

Not surprisingly, ATF1 also contributes to vegetative growth. In accordance with this, reduced vegetative growth has been reported in the *ΔFvatfA*‐mutant strain of *Fusarium verticillioides* ^44^ and the *ΔMoatf1*‐mutant strain of *Magnaporthe oryzae* ^45^. In *A. nidulans* and *A. alternata*, blue light markedly inhibits conidial germination^34^, ^46^. However, conidial germination in *T. guizhouense* is promoted by constant blue light illumination. More surprisingly, conidia in the *Δhog1*‐mutant strain germinated ahead of time in the dark, suggesting that HOG1 has a repressive role in conidial germination. Inversely, conidial germination of the *Δatf1*‐mutant strain was remarkably delayed, as reported for mutants of its orthologs in other fungi^47^, ^48^. The conidial germination ratio was significantly reduced in the *A. oryzae ΔatfA*‐mutant because of decreased glutamate levels in conidia^47^. Notably, in both the *Δhog1*‐ and *Δatf1*‐mutant strains, the conidia germinated earlier under constant blue light than in the dark. It seems that HOG1 and ATF1 take part in the maintenance of normal conidial germination but are not directly involved in the light‐induced promotion of conidial germination. In addition, it is worth noting that *atf1* expression was not significantly affected in the *Δhog1*‐mutant strain under blue light (Figure 1A), suggesting that the enhanced conidial germination in the *Δhog1*‐mutant strain is independent of ATF1.

Under H_2_O_2_ stress, the absence of HOG1 and ATF1 negatively affected mycelial growth and conidial viability in *T. guizhouense*. Moreover, compared with the *Δhog1*‐mutant strain, the *Δatf1*‐mutant strain was more sensitive to oxidative stress. In *A. nidulans*, SakA and AtfA are essential for the resistance of conidia to H_2_O_2_ stress but not for the resistance of mycelia^5^, ^35^. Like in *A. nidulans*, conidia of *T. guizhouense* were more sensitive to H_2_O_2_ than mycelia. Our previous study showed that in *T. guizhouense*, blue light effectively enhances conidial resistance to oxidative stress by inducing the expression of stress‐related genes, such as catalase‐encoding genes^33^. The results of the present study show that the expression of catalase‐encoding genes under blue light is strictly regulated by HOG1 and ATF1. The BiFC assay demonstrated that *T. guizhouense* HOG1 physically interacts with ATF1 in response to H_2_O_2_ stress, consistent with the results of a previous study on *A. nidulans* ^5^. Likewise, blue light can activate the interaction between HOG1 and ATF1. Therefore, it is likely that all signals that activate the HOG pathway are able to promote this interaction. This further increases the complexity of signal discrimination for the HOG pathway.

Our results suggest that *T. guizhouense* is more sensitive to blue light than other *Trichoderma* species. In the present study, 824 DEGs were identified after 30 min of blue light illumination, and in our previous study, 1615 DEGs were identified after 45 min of blue light illumination^28^. In comparison, only 135 blue light‐responsive genes were identified in *T. atroviride* ^29^. The genome‐wide transcriptome analyses of *T. guizhouense* in this study show that 61.8% of the blue light‐regulated genes in the wild type are controlled by ATF1, and after blue light exposure, 58.4% of DEGs in the wild type are regulated by both HOG1 and ATF1, confirming their critical roles in blue light signaling. In *A. nidulans*, AtfA is essential for red light activation of *ccgA* and *conJ* ^13^. Analyses of transcriptome data of *A. nidulans* revealed that AtfA also regulates many stress‐unrelated genes^6^, ^49^. In the present study, the deletion of ATF1 caused differential expression of 584 genes in the dark, of which 439 were downregulated. That is, ATF1 maintains the basal expression of a large number of genes under moderate conditions without any stimuli. It is also worth mentioning that SakA in *A. nidulans* controls the basal expression of more genes^22^. In *T. guizhouense*, only 19.0% (195 out of 1024) of genes controlled by HOG1 overlapped with those regulated by ATF1 under conditions without stimuli, suggesting that ATF1 and HOG1 have distinct functions. This explains why they play opposite roles in conidial germination.

**Figure 6:**
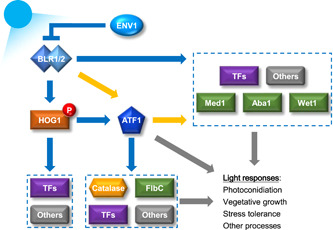
Regulatory network of ATF1 in response to blue light signaling in *Trichoderma guizhouense*.

The schematic model of blue light signaling in *T. guizhouense* proposed previously^28^ is updated in Figure 6. The BLR1/2 complex perceives the blue light signal and activates the MAPK HOG pathway. ENV1 inhibits the activity of the BLR1/2 complex. In the nucleus, phosphorylated HOG1 interacts with ATF1, which activates the expression of genes encoding TFs. The blue light signal is transmitted from HOG1 to ATF1 and further activates the expression of genes encoding catalase, FlbC, and other TFs. ATF1 also regulates the expression of *med1*, *aba1*, *wet1*, and some TF‐encoding genes in response to blue light, independent of HOG1. Overall, the results of our study demonstrate that ATF1 plays crucial roles in blue light signaling and physiological responses in *T. guizhouense*.

## MATERIALS AND METHODS

### Strains and culture conditions

The wild type and mutant strains of *T. guizhouense* NJAU4742 (*T. guizhouense*) were cultured with PDA and broth (PDB) media (BD Difco) at 28°C under dark and blue light (1.7 μmol photons/(m^2^ × s)) conditions in self‐designed light boxes described previously^22^. *Escherichia coli* DH5α, used for plasmid propagation and maintenance, was cultured in Luria–Bertani medium (LB; Sangon Biotech) containing 100 μg/ml ampicillin. The strains used in this study are listed in Table S1.

### Construction of mutant strains

Transformation of *T. guizhouense* was performed according to the protocol established previously^50^. For the construction of the *atf1*‐deletion mutant, ~1.5 kb upstream and downstream fragments of *atf1* were amplified and fused with hygromycin phosphotransferase gene (*hph*) cassette^51^. Positive transformants were verified by PCR and Southern blot. To construct the re‐complemented strain, the geneticin (G418)‐resistance gene and the full‐length *atf1* gene were amplified and cloned into the vector pUC19 using the ClonExpress® MultiS One Step Cloning Kit (Vazyme Biotechnology Co., Ltd.). The plasmid was ectopically transformed into the *atf1* mutant, and the integrity of the insert was confirmed by PCR. To construct the mutant with an overexpression allele of ATF1, *hph* cassette, the *T. reesei cdna1* promoter, and the full‐length *atf1* gene were amplified and cloned into the vector pUC19. The plasmid was ectopically transformed into the wild type, and the expression level of *atf1* was confirmed by RT‐qPCR. The *hog1*‐deletion mutant strain construction was described in a previous study^28^.

To tag ATF1 with green fluorescent protein, ORF of *atf1*, *egfp* with a synthetic linker (3× GGGGS), *hph* cassette, and ~1.5 kb downstream fragments of *atf1* were amplified separately and fused in order. PCR products were used for transformation, and the positive transformants were verified by PCR.

To visualize the interaction between HOG1 and ATF1 using the bimolecular fluorescence complementation (BiFC) assay, two proteins were tagged with split yellow fluorescent protein (YFP). Fragments encoding the N‐terminal half of YFP (YFP‐N) and the C‐terminal half of YFP (YFP‐C) were amplified from the plasmids pZY25 and pJP5, respectively. To fuse the YFP‐N to ATF1, *hph* cassette, *cdna1* promoter, *YFP‐N* with the synthetic linker (RSIAT), and the entire ORF of *atf1* were amplified and cloned into the vector pUC19, yielding the plasmid pYL01 (*hph*::*cdna1*(*p*)::*YFP‐N*::*atf1*). To tag the HOG1 with YFP‐C, the G418‐resistance gene, *cdna1* promoter, *YFP‐C* with the synthetic linker (RPACKIPNDLKQKVMNH), and the entire ORF of *hog1* were amplified and cloned into the vector pUC19, yielding the plasmid pYL02 (G418::*cdna1*(*p*)::*YFP‐C*::*hog1*). The plasmids pYL01 and pYL02 were transformed together or separately into the wild type ectopically and the integrity of the insert was confirmed by PCR. All primers used in this study are listed in Table S2.

### Microscopy

To analyze the subcellular localization of ATF1, fresh conidia of the strain ATF1::eGFP were inoculated on microscope coverslips with 400 µl supplemented PDB medium and cultured at 28°C in the dark for 14 h. In BiFC assay, fresh mycelia were cultured on PDA plates at 28°C in the dark. After 24 h culture, samples were exposed to blue light for 3 min. To impose oxidative stress, samples were treated with 1× phosphate‐buffered saline containing 10 mM hydrogen peroxide for 3 min. Nuclei were stained with Hoechst (no. C0031; Solarbio) before microscopy with a fluorescence microscope Leica DM2000 (Germany).

### Quantification of conidia

For the quantification of conidia, fresh mycelia of *T. guizhouense* strains were inoculated on PDA plates (Ø 6 cm) and cultured in the dark or constant blue light for 3 days. Conidia were collected with 20 ml distilled water and the suspension was filtered through Miracloth (Millipore, Merck KGaA). Conidia concentration was determined using a hemocytometer.

### Assessment of conidial germination

Conidia of different strains produced in blue light were harvested as described above and conidial suspensions of each strain were diluted to a final concentration of 1 × 10^6^ spores/ml. 1 × 10^5^ conidia of each strain were inoculated on coverslips with 400 µl PDB medium and cultured at 28°C under dark and blue light conditions. Germination rates were determined under the microscope after 12 h culture. As for the oxidative stress, conidia were cultured in PDB medium supplemented with 1.5 mM hydrogen peroxide in the dark.

### Measurement of fungal growth rate

To measure the growth rate of each strain under dark and blue light conditions, each strain was cultured for 2 days. Colony diameters of each strain were measured after 48 h culture. To analyze the resistance of mycelia to oxidative stress, fresh mycelia of each strain were inoculated on PDA plates (Ø 6 cm) in the presence or absence of 30 mM hydrogen peroxide. To evaluate conidial resistance, conidia of each strain were cultured on PDA medium (Ø 6 cm Petri dish) with or without 3 mM hydrogen peroxide. The growth inhibition rate of each strain was calculated after 2 days of culture at 28°C in the dark. Assessment of conidia viability was performed as previously described^33^, ^52^. One hundred microliters of conidial suspension (1 × 10^3^ conidia/ml) of each strain was spread on PDA medium (Ø 9 cm Petri dish) containing 3 mM hydrogen peroxide or not. Then the plates were cultured in the dark at 28°C for 30 h.

### Transcriptome analysis

Fresh mycelia of the wild type and *Δhog1*‐ and *Δatf1*‐mutant strains were inoculated on cellophane‐covered PDA plates (Ø 6 cm) at 28°C in the dark. After 24 h growth, all strains were kept in the dark or exposed to blue light for 30 min. Then mycelia were harvested in dim‐green light and frozen immediately using liquid nitrogen. Three biological replicates were prepared for each condition. The transcriptome sequencing was performed at Gene Denovo Biotechnology Co., Ltd. using the Illumina HiSeq. 2500 platform. After being filtered by fastp (version 0.18.0), clean reads were mapped to the reference genome of *T. guizhouense* using HISAT2.2.4. Gene expression levels were calculated with the fragment per kilobase of transcript per million mapped reads (FPKM) value, using StringTie v1.3.1. DEGs were identified using DEGseq. 2 software with thresholds of false discovery rate (FDR) < 0.05 and absolute fold change ≥2. The function of the DEGs was predicted using KEGG (https://www.genome.jp/kegg/) and GO (https://www.geneontology.org/) databases. The sequencing data are available in the NCBI database Sequencing Read Archive (SRA) under the accession number PRJNA937411.

### RNA isolation and RT‐qPCR

For sample collection, all strains were cultured on cellophane‐covered PDA plates (Ø 6 cm) at 28°C. To evaluate the effect of illumination time on gene expression, the wild type was grown in the dark for 24 h, which was then exposed to blue light for 5, 10, 15, 30, 45, 60, 90, and 120 min. To evaluate the relative expression of specific genes in different mutant strains after short‐time illumination, strains were cultured in the dark for 24 h before being exposed to blue light for 30 min. For constant blue light illumination, all strains were cultured in the dark or in blue light for 48 h. Samples were harvested in dim green light after different light treatments and immediately frozen using liquid nitrogen. Three biological replicates were prepared for each condition. Total RNA extraction, cDNA synthesis, and RT‐qPCR assay were performed as previously described^33^. The relative expression of each gene was normalized to the translation elongation factor 1 alpha (*tef1*) gene (OPB38715).

### DNA extraction and Southern blot analysis

Fresh mycelia were inoculated on PDA plates covered with cellophane and cultured in the dark at 28°C for 4 days. Afterward, mycelia were collected and ground in liquid nitrogen, and the DNA was extracted using the Fungal DNA kit (OMEGA) according to the manufacturer's instruction. Standard protocols were used for electrophoresis and the Southern blot hybridization was carried out using the Digoxigenin High Prime DNA Labeling and Detection Starter Kit I (Roche) according to the manufacturer's instruction.

### Statistical analysis

Data are presented as means ± standard deviation (SD) from three biological replicates. The statistical analysis was carried out in the IBM SPSS Statistics 25 program. One‐way analysis of variance with the Bonferroni correction was used when one sample is repeatedly compared to other samples in the same experiment, and different lowercase letters represent significant difference (*p* < 0.05). For simple pairwise comparison, Student's *t*‐test was used for statistical analysis (*p < 0.05, **p < 0.01, ***p < 0.001, ns, not significant). Venn diagram and heatmap were generated in the R environment.

## AUTHOR CONTRIBUTIONS

**Yifan Li**: Conceptualization (equal); data curation (lead); formal analysis (lead); investigation (lead); writing—original draft (lead). **Yanshen Li**: Investigation (equal). **Huanhong Lu**: Investigation (equal). **Tingting Sun**: Data curation (supporting); formal analysis (supporting). **Jia Gao**: Formal analysis (supporting). **Jian Zhang**: Methodology (supporting); project administration (supporting). **Qirong Shen**: Conceptualization (equal). **Zhenzhong Yu**: Conceptualization (lead); data curation (equal); formal analysis (equal); funding acquisition (lead); project administration (equal); supervision (lead); writing—review and editing (equal).

## ETHICS STATEMENT

No animals or humans were involved in this study.

## CONFLICT OF INTERESTS

The authors declare no conflicts of interest.

## Supporting information

Supporting information.

Supporting information.

Supporting information.

## Data Availability

The data that support the findings of this study are available from the corresponding author upon reasonable request.
