# Supplementary material for: The bZIP transcription factor ATF1 regulates blue light and oxidative stress responses in Trichoderma guizhouense
Source: mLife. 2023 Dec 4;2(4):365–77. doi: 10.1002/mlf2.12089 (PMC10989065; doi:10.1002/mlf2.12089)

Figure S1. Sequence analysis of ATF1 in *T. guizhouense*.

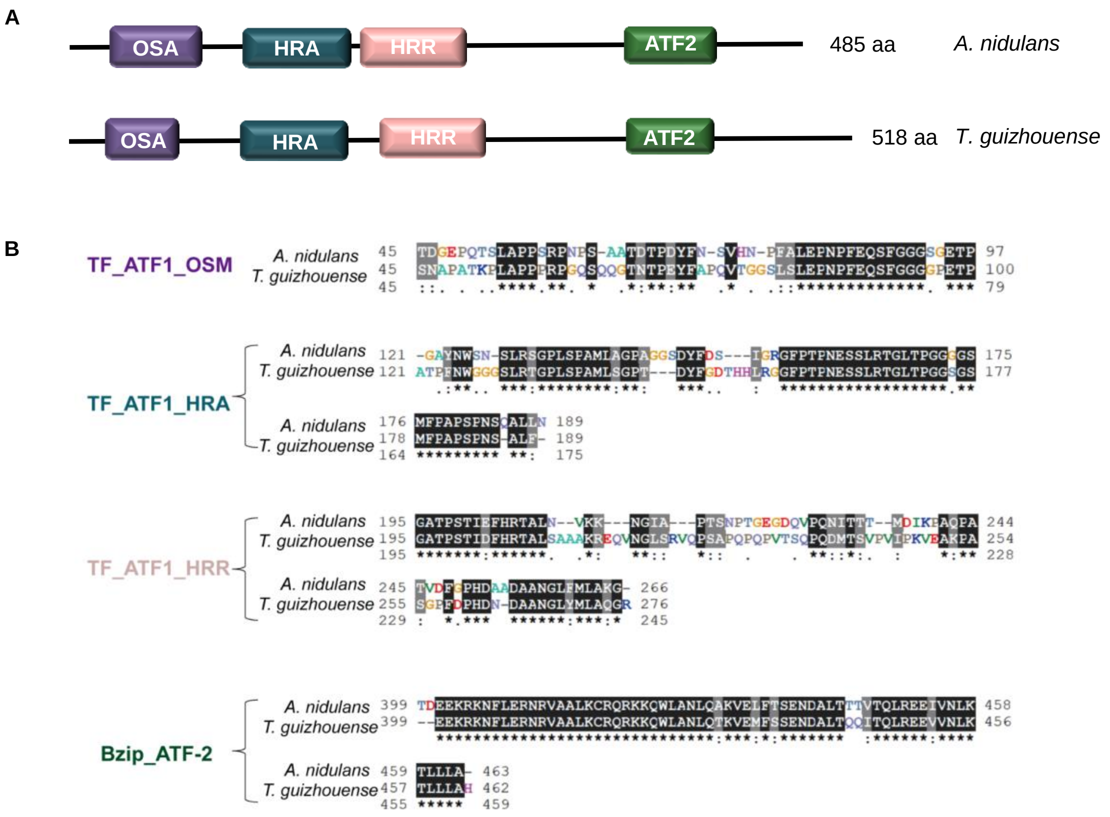

Figure S2. Verification of the ATF1-encoding gene deletion mutant.

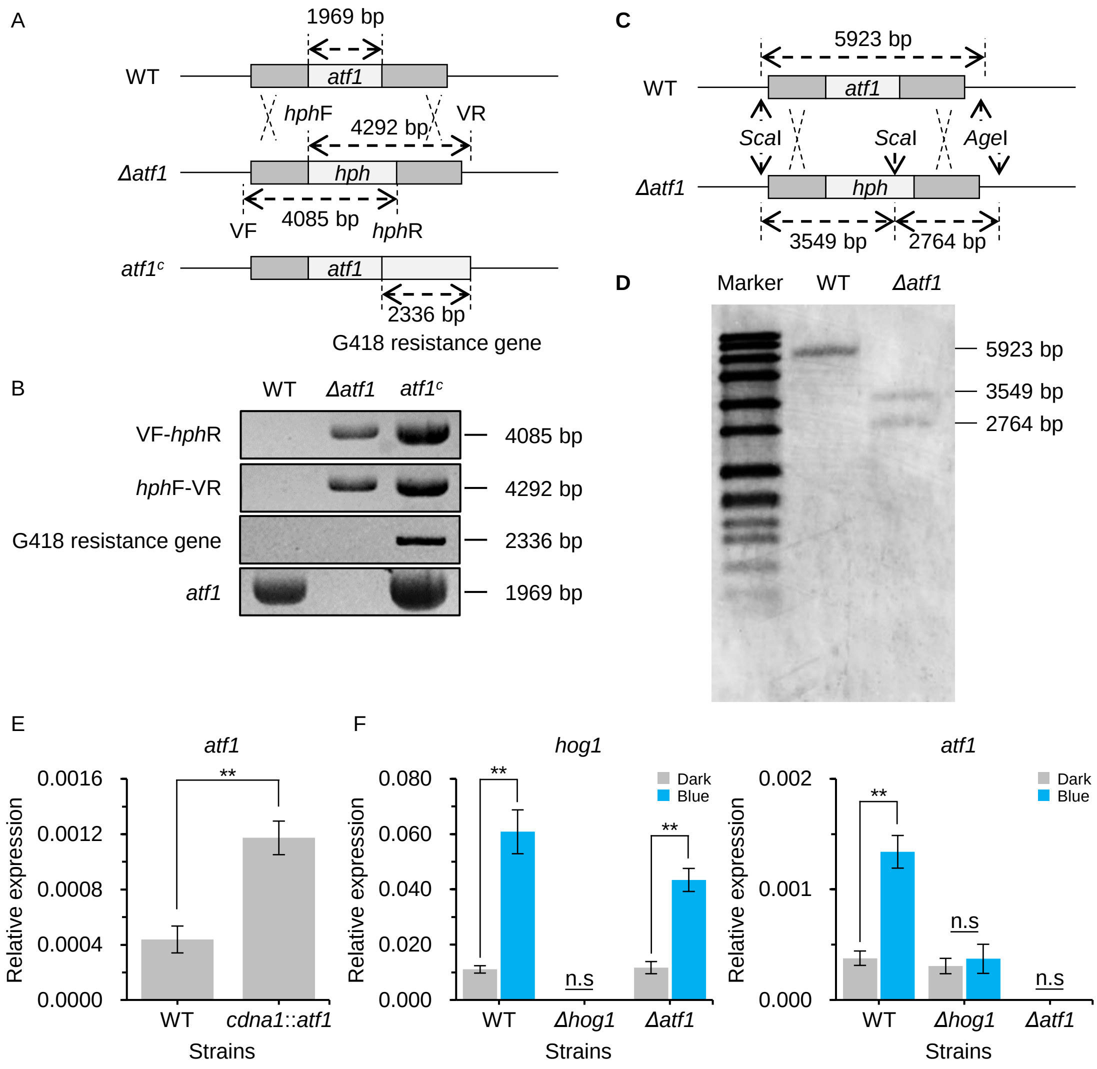

Figure S3. Enriched GO terms and KEGG pathways of DEGs in *Δhog1*- and *Δatf1*-mutant strains in the dark.

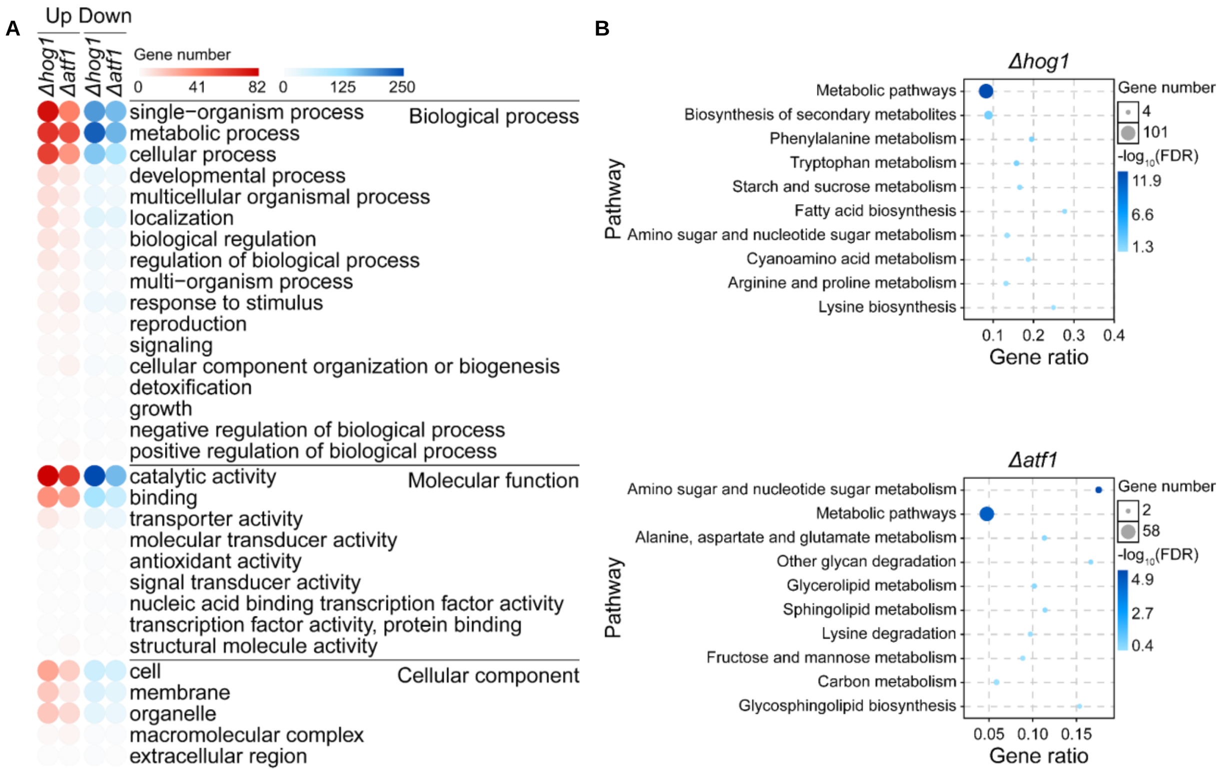

Figure S4. Differentially expressed TF-encoding genes identified in *Δhog1*-, and *Δatf1*-mutant strains in the dark.

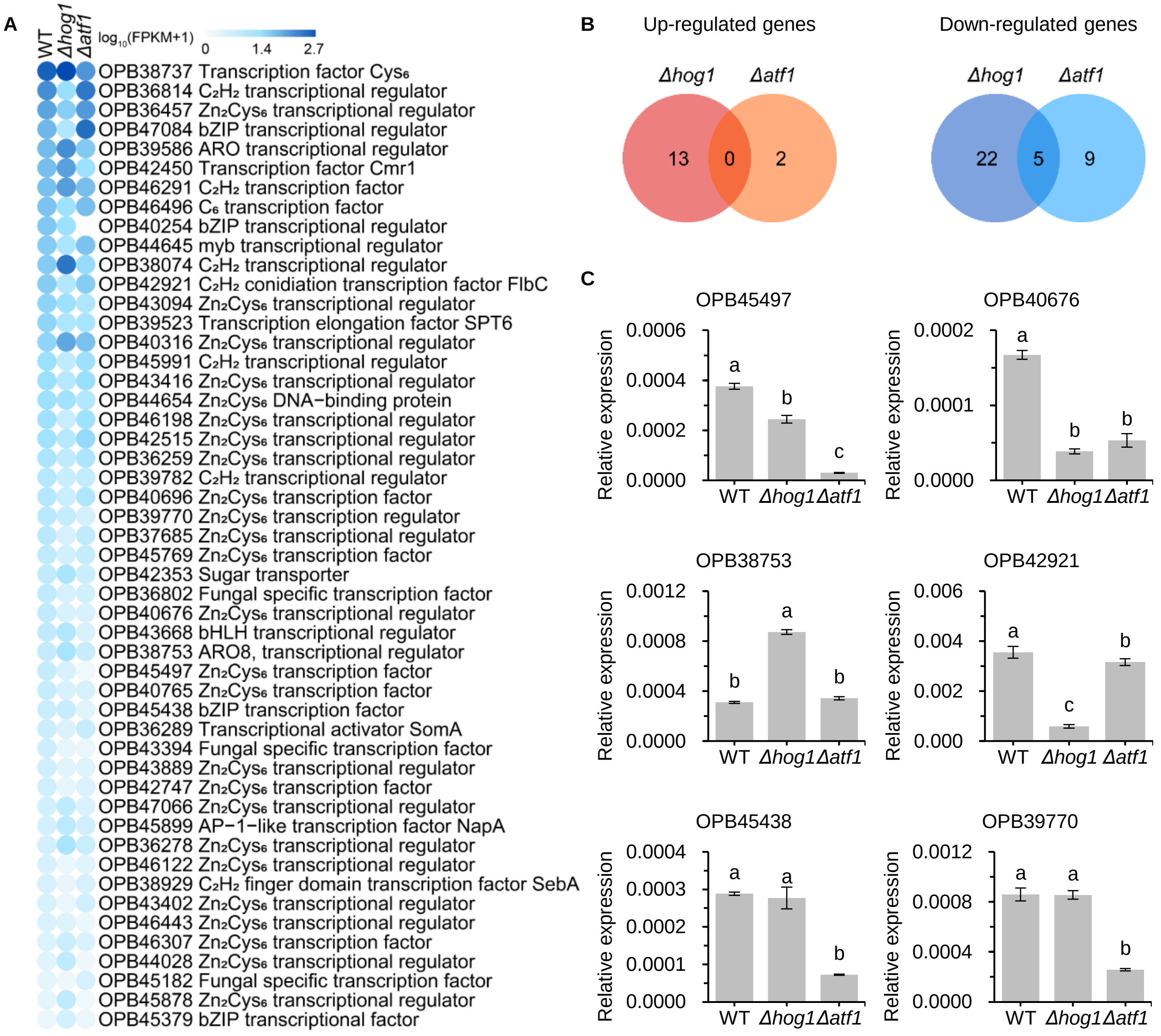

Supplement: Supplementary file 1 — Supporting information. [file MLF2-2-365-s001.pdf]
