# Supplementary material for: The bZIP transcription factor ATF1 regulates blue light and oxidative stress responses in Trichoderma guizhouense
Source: mLife. 2023 Dec 4;2(4):365–77. doi: 10.1002/mlf2.12089 (PMC10989065; doi:10.1002/mlf2.12089)
Supplement: Supplementary file 3 — Supporting information. [file MLF2-2-365-s002.docx]

**Supplementary Figures**

**Figure S1.** Sequence analysis of ATF1 in *T. guizhouense*.

(A) Conserved protein domain prediction of AtfA in *A. nidulans* and ATF1 in *T. guizhouense*. (B) Alignment of conserved structural domains of ATF1 and AtfA using Bioedit software.

**Figure S2.** Verification of ATF1-encoding gene deletion mutant.

(A) Construction and verification strategy for *Δatf1*- and *atf1^c^*-mutant strains. (B) PCR verification of *Δatf1*- and *atf1^c^*-mutant strains. (C) Verification strategy used for Southern blot analysis. (D) Southern blot analysis of wild type and *Δatf1*-mutant strains. (E) Transcript levels of *atf1* in wild type and *pcdna1*::*atf1*-mutant strain. All strains were grown on PDA medium at 28 °C in the dark. (F) Transcript levels of *atf1* and *hog1* in wild type, and *Δhog1*-, and *Δatf1*-mutant strains. Transcript level of each gene was normalized to that of *tef1*. Data represent mean ± SD (*n* = 3). Data were analyzed by *t*-test (*p** < 0.05, *p*** < 0.01, *p**** < 0.001, ns, not significant).

**Figure S3.** GO terms and KEGG pathways significantly enriched with DEGs in *Δhog1*- and *Δatf1*-mutant strains in the dark.

(A) GO enrichment analysis of DEGs in *Δhog1*- and *Δatf1*-mutant strains in the dark. (B) Top 10 KEGG pathways significantly enriched with DEGs identified in *Δhog1*- and *Δatf1*-mutant strains in the dark. Color of circles indicates value of -log_10_(FDR). Gene ratio refers to ratio of number of DEGs to total number of genes in the pathway.

**Figure S4.** Differentially expressed TF-encoding genes identified in *Δhog1*- and *Δatf1*- mutant strains in the dark.

(A) Transcriptional profiles of differentially expressed TF-encoding genes identified in *Δhog1*- and *Δatf1*-mutant strains. (B) Venn diagrams of differentially expressed TF-encoding genes in *Δhog1*-, and *Δatf1*-mutant strains. (C) Transcript levels of selected TF-encoding genes in wild type and *Δhog1*- and *Δatf1*-mutant strains. All strains were grown on PDA plates at 28 °C in the dark for 24 h. Transcript level of each gene was normalized to that of *tef1*. Error bars indicate SD of three biological replicates. Data were analyzed by one-way ANOVA with Bonferroni correction. Different lowercase letters represent significant difference (*p* < 0.05).
